# Assessment of brain injury characterization and influence of modeling approaches

**DOI:** 10.1038/s41598-022-16713-2

**Published:** 2022-08-10

**Authors:** Saichao Yang, Jisi Tang, Bingbing Nie, Qing Zhou

**Affiliations:** State Key Lab of Automotive Safety and Energy, School of Vehicle and Mobility, Tsinghua University, Beijing, 100084 China

**Keywords:** Biophysics, Engineering, Mathematics and computing

## Abstract

In this study, using computational biomechanics models, we investigated influence of the skull-brain interface modeling approach and the material property of cerebrum on the kinetic, kinematic and injury outputs. Live animal head impact tests of different severities were reconstructed in finite element simulations and DAI and ASDH injury results were compared. We used the head/brain models of Total HUman Model for Safety (THUMS) and Global Human Body Models Consortium (GHBMC), which had been validated under several loading conditions. Four modeling approaches of the skull-brain interface in the head/brain models were evaluated. They were the original models from THUMS and GHBMC, the THUMS model with skull-brain interface changed to sliding contact, and the THUMS model with increased shear modulus of cerebrum, respectively. The results have shown that the definition of skull-brain interface would significantly influence the magnitude and distribution of the load transmitted to the brain. With sliding brain-skull interface, the brain had lower maximum principal stress compared to that with strong connected interface, while the maximum principal strain slightly increased. In addition, greater shear modulus resulted in slightly higher the maximum principal stress and significantly lower the maximum principal strain. This study has revealed that using models with different modeling approaches, the same value of injury metric may correspond to different injury severity.

## Introduction

Brain injuries exhibit high incidence and high mortality in vehicle collision accidents. An investigation from Centers for Disease Control and Prevention in American showed that there were about 2.87 million Traumatic Brain Injury (TBI) related emergency department (ED) visits, hospitalizations, and deaths occurred in the United States in 2014, with an average of 155 people died each day from injuries that include TBI^1^. Falls (52%) and motor vehicle crashes (20%) were the first and second leading causes of all TBI-related hospitalizations^1^. Based on NASS-CDS analyses of frontal crashes^2^, fatalities attributable to head injuries, with societal costs exceeding $6 Billion, are second only to fatalities attributable to thoracic region. TBI in vehicular accidents is one of the major causes of mortality and permanent disability in many countries^3^. For instance, in the USA, such accidents account for the greatest fraction (31.8%) of the TBI-related deaths^3^. Comprehensive understanding to brain injury may improve restraint system and traffic safety.

Brain injuries have different forms and severities, with different mechanisms and physiological characterizations. Understanding injury mechanisms may enhance the injury characterization in brain models. Clinically brain injuries can be classified in two broad categories: diffuse injuries and focal injuries. Statistics showed that 3/4 automobile/pedestrian brain injuries were of diffuse type, while assault and fall victims had 3/4 focal injuries^4^. Acute subdural hematoma (ASDH) and diffuse axonal injury (DAI) were the two most important causes of death, which accounted for more head injury deaths than all other lesions combined^4^. DAI is regarded as one of the most severe brain diffuse injuries, caused by axon damage in brain and the degrees of morbidity depend on the number of axons that are damaged^5,6^. As strain level applied to axons grows in intensity, a series of increasingly severe pathological changes occur. At strain levels of 10–15%, axonal swelling and a total loss of axonal transport begin (2–6 h after the injury)^7^. Hematoma is a typical brain focal injury, classified by injury region and includes epidural hematoma (EDH), subdural hematoma (SDH), and intracerebral hematoma (ICH). Subdural hematoma is caused by penetrating wounds, large-contusion bleeding into the subdural, or more commonly tearing of veins that bridge the subdural (called bridging veins, BVs). According to Gennarelli and Thibault^4^, ASDH is the most important cause of death in severely head-injured patients due to high incidence (30%), high mortality (60%), and head injury severity (2/3 with Glasgow Coma scores of 3–5).

Finite element (FE) model of human head is suitable tool for studying the complex, tissue-level mechanical response of brain injury in head impact. From 1975, after the first head and brain FE model was proposed by Shugar and Katona^8^, many FE brain models have been developed and used for impact induced injury analysis. A few widely recognized brain models include WSUBIM (Wayne State University Brain Injury Model)^9^, SIMon (by University of Western Australia)^10^, KTH (by Royal Institute of Technology Sweden)^11^, UCDBTM (by Department of Mechanical Engineering, University College Dublin, Ireland)^12,13^, SUFEHM (Strasbourg University Finite Element Head Model)^14–16^, and the head part from whole body FE models such as THUMS (Total Human Model for Safety)^17^ and GHBMC (Global Human Body Models Consortium)^18^, etc. Most brain tissues are reflected in those models to characterize specific forms of injury. For example, bridging vein (BV) is a key component for research on ASDH. On human brain, one end of the bridging vein is connected to the cerebrum and the other end is to the superior sagittal sinus (SSS). The BVs in some FE brain models were simplified to spring or beam elements. In recent years, with the development of new imaging technique (e.g. Fiber Tractograghy), more detailed parts in brain have been modeled, such as axonal fibers^19–21^, the injury of which is related with DAI. Zhao et al.^22^ has generated vascular meshes to study the relation between cerebral vascular strains and injury.

**Figure 1:**
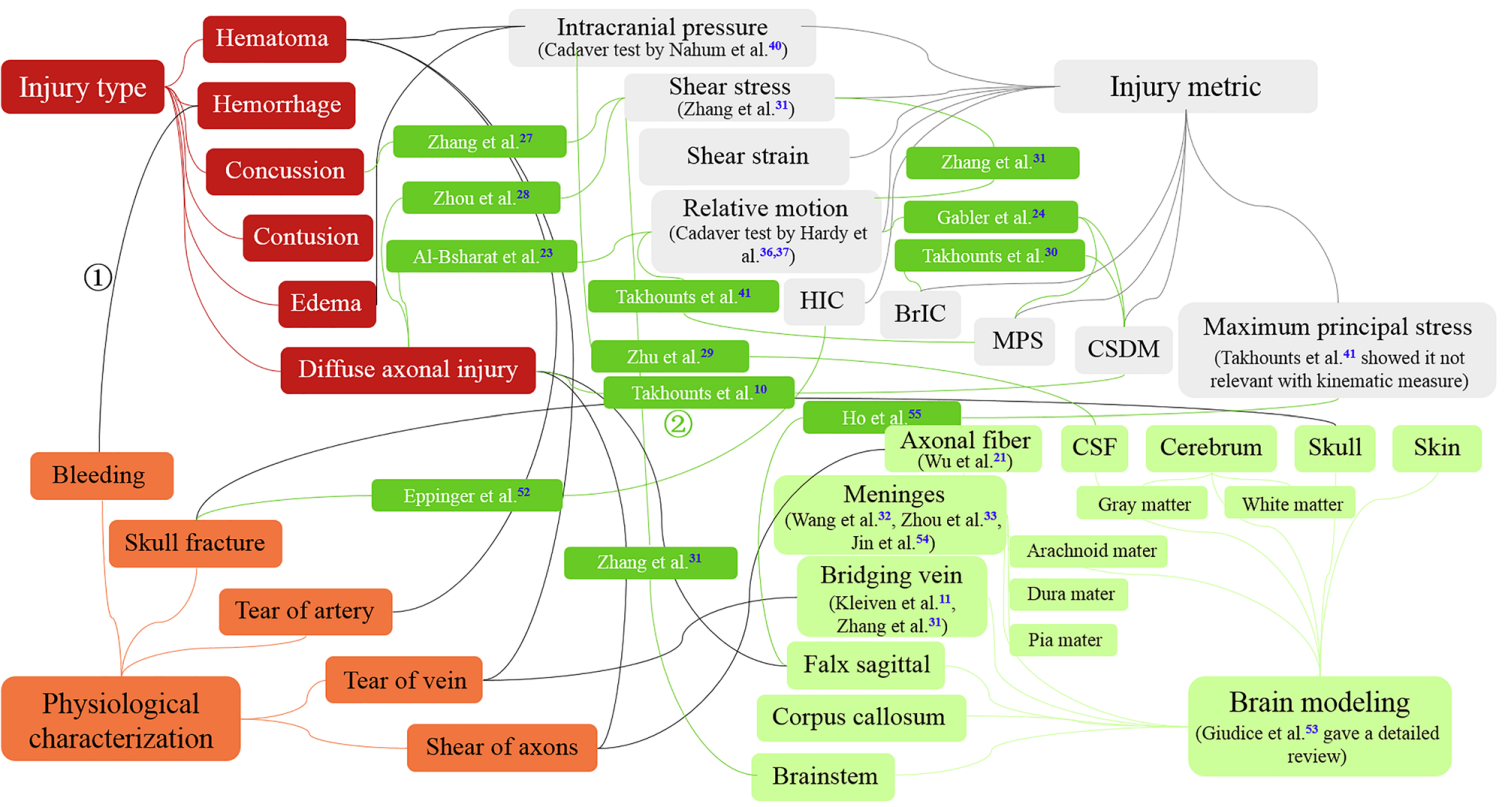
Existing research or medical knowledge on correlation among injury type, physiological characterization, brain modeling, and injury metric.

Using brain models, some efforts were put on developing brain injury metrics, which is used for injury characterization or prediction with outputs in brain models. As summarized in Fig. 1, there are two representative research approaches. The counterclockwise flow shows a routine and logical way. It first studies physiological characterization of common brain injury types based on medical knowledge, then builds related brain structures in FE models, and lastly, uses physical parameters or physical phenomenon such as structural fracture to characterize the corresponding injury. The modeling of BV and axonal fibers are representatives of such an approach. However, the research progress using this approach is relatively limited and slow, as using these structures to represent brain injury requires advanced imaging technology and detailed biological material information. Takhounts et al. (2003) pointed out that while explicit BVs were modeled in SIMon, they were not used in injury prediction. In contrast, more researches have been done in direct and more effective way. They establish correlations between brain injury and corresponding injury metrics using regression method or machine learning method^10,23–29^ or between different metrics^24,30,31^. Each line in Fig. 1 indicates a piece of medical knowledge or some existing researches. For example, “line 1” indicates that bleeding happens when suffering a hemorrhage, while “line 2” represents that Takhounts et al.^10^ have studied the relation between DAI and Cumulative Strain Damage Measure (CSDM).

As the brain models were developed by various organizations, there were differences in modeling approaches. The two main aspects are material property and structure definition. THUMS, SIMon and GHBMC use linear viscoelastic material models for brain tissue, while KTH and SUFEHM use hyperelastic-viscoelastic models. The properties assigned to these materials vary from the stiffest with G0 = 49 kPa and G1 = 16 kPa (SUFEHM) to the softest with G0 = 1.6 kPa and G1 = 0.9 kPa (SIMon). G0 and G1 are short-time and long-time (infinite) shear modulus. The contact definitions between the parts are different as well. Wang et al.^32^ studied four kinds of interface between skull and brain, from the strongest (directly attaching to each other) to the weakest (friction-less sliding contact). THUMS and GHBMC represents two typical contact definitions, as the skull in THUMS is connected to brain with solid viscoelastic CSF elements while a gap is kept between the skull and brain in GHBMC. Zhou et al.^33^ modified the CSF elements in KTH using ALE multi-material formulation to model the fluid properties of it. There have been debates in the academic circle about the definitions of the skull-brain interface. Different FE modeling approaches affect mechanical responses^9,32,34,35^, and therefore, validation of model is important in developing human body model (HBM) to ensure the biofidelity. To validate the FE brain models, researchers usually used brain motion or intracranial pressure from cadaver tests^36–38^. The head models in THUMS and GHBMC, as two of the commonly used HBMs in our field, have been validated under a variety of loading conditions in their respective development processes, including using the same loading conditions such as the mechanical response of skull^39^, intracranial pressure^40^, and displacement of internal brain markers^36^. Those validations have ensured similar mechanical responses of the two models.

Although most brain models have been validated under several similar load cases, different modeling approaches can lead to different kinematic and kinetic response in application, even different injury metric values. The influence of modeling approaches on injury metric were less considered in the literature. Takhounts et al.^10,41^ published two papers in 2003 and 2008, respectively, studying the correlations of biomechanical injury metrics with their corresponding injuries. Surprisingly, they found that the correlation between RMDM (relative motion damage measure) and ASDH was totally different using different versions of SIMon models. It means that output of injury metrics are highly dependent on the models. Even differences between model versions could matter. These issues have increased the complexity and unknowingness of the problem. In this study, we compared the kinematic and mechanical responses of four brain models with different modeling approaches, concentrating on brain-skull interface and brain material property. The loading condition was based on animal tests from Abel et al.^42^.The influence of brain modeling approaches on the application of injury metrics was also discussed.

## Methods

This section describes the model generation and loading condition in the simulations. Two validated head/brain models were selected as the baseline model. They were extracted from the THUMS AM50 occupant model (version 4.02) and GHBMC M50-Occupant model (version 4.5). Then two additional models were generated by changing the brain-skull interface and material property of cerebrum of the THUMS head model. Sagittal plane rotation in Abel’s animal test were selected as load cases to compare simulation results with the DAI and ASDH injury results in the animal test. Three injury metrics, maximum principal stress, maximum principal strain and CSDM, were assessed.

### Model description

The anatomical structures of THUMS and GHBMC are similar, both comprising of skin, skull, meninges (dura mater, arachnoid mater, pia mater), cerebrospinal fluid (CSF), and cerebrum (gray matter and white matter), while the geometry of each part is slightly different. Table 1 tabulates the FE settings of the brain components of the two models, including the element type, material card and parameters. THUMS and GHBMC use similar material properties on cerebrum for gray matter and white matter. The contact definitions between skull and brain (i.e., contact between meninges) are different. In THUMS, the dura and arachnoid are tied by a layer of solid elements representing the CSF in between, while in GHBMC, it is modeled as separate surfaces with a 0.1 mm gap and regarded as a “sliding interface” with contact friction coefficient of 0.1.

**Table 1:** Key tissues and anatomical components of brain models in THUMS and GHBMC.

Part	THUMS					GHBMC				
	Name	Element type	Material			Name	Element type	Material		
			Name	Density (kg/m^3^)	Modulus (MPa)			Name	Density (kg/m^3^)	Modulus (MPa)
Skin	Skin_out	Shell	34-Fabric	1100	EA = 22 EB = 0	Skin	Shell	1-Elastic	1100	E = 10
	Skin	Solid	181-Simplified_Rubber	1050	K = 4.59	Scalp	Solid	6-Viscoelastic	1100	K = 20
	Skin_in	Shell	34-Fabric	850	EA = 22 EB = 0					
Skull	Skull_external	Shell	81-Plasticity_with_damage	2120	E = 14,900	Skull_outer	Solid	24-Piecewise_linear_Plasticity	2100	E = 10,000
	Skull	Solid	105-Damage_2	1000	E = 1090	Skull_diploe	Solid	24-Piecewise_linear_Plasticity	1000	E = 600
	Skull_internal	Shell	81-Plasticity_with_damage	2120	E = 10,900	Skull_inner	Solid	24-Piecewise_linear_Plasticity	2100	E = 10,000
Meninges	Dura	Shell	1-Elastic	1130	E = 31.5	Dura	Shell	1-Elastic	1100	E = 31.5
	CSF2	Solid	6-Viscoelastic	1000	K = 2000	Gap				
	Arachnoid	Shell	1-Elastic	1000	E = 1.1	Arachnoid	Shell	1-Elastic	1100	E = 12
	CSF	Solid	6-Viscoelastic	1000	K = 2000	CSF_Cerebrum	Solid	61-Kelvin-Maxwell_Viscoelastic	1040	K = 2190
	Pia	Shell	1-Elastic	1000	E = 1.1	Pia	Shell	1-Elastic	1100	E = 12.5
Cerebrum	Gray_matter	Solid	61-Kelvin-Maxwell_Viscoelastic	1000	K = 2160; G0 = 0.006; G1 = 0.0012	Gray_matter	Solid	61-Kelvin-Maxwell_Viscoelastic	1060	K = 2190; G0 = 0.006; G1 = 0.0012
	White_matter	Solid	61-Kelvin-Maxwell_Viscoelastic	1000	K = 2160; G0 = 0.006; G1 = 0.0012	White_matter	Solid	61-Kelvin-Maxwell_Viscoelastic	1060	K = 2190; G0 = 0.0075; G1 = 0.0015

Notions: *E* Young’s modulus, *EA, EB* Young’s modulus in longitudinal and transverse direction for Fabric material, *K* bulk modulus, *G0, G1* short-time and long-time shear modulus for viscoelastic material.

**Figure 2:**
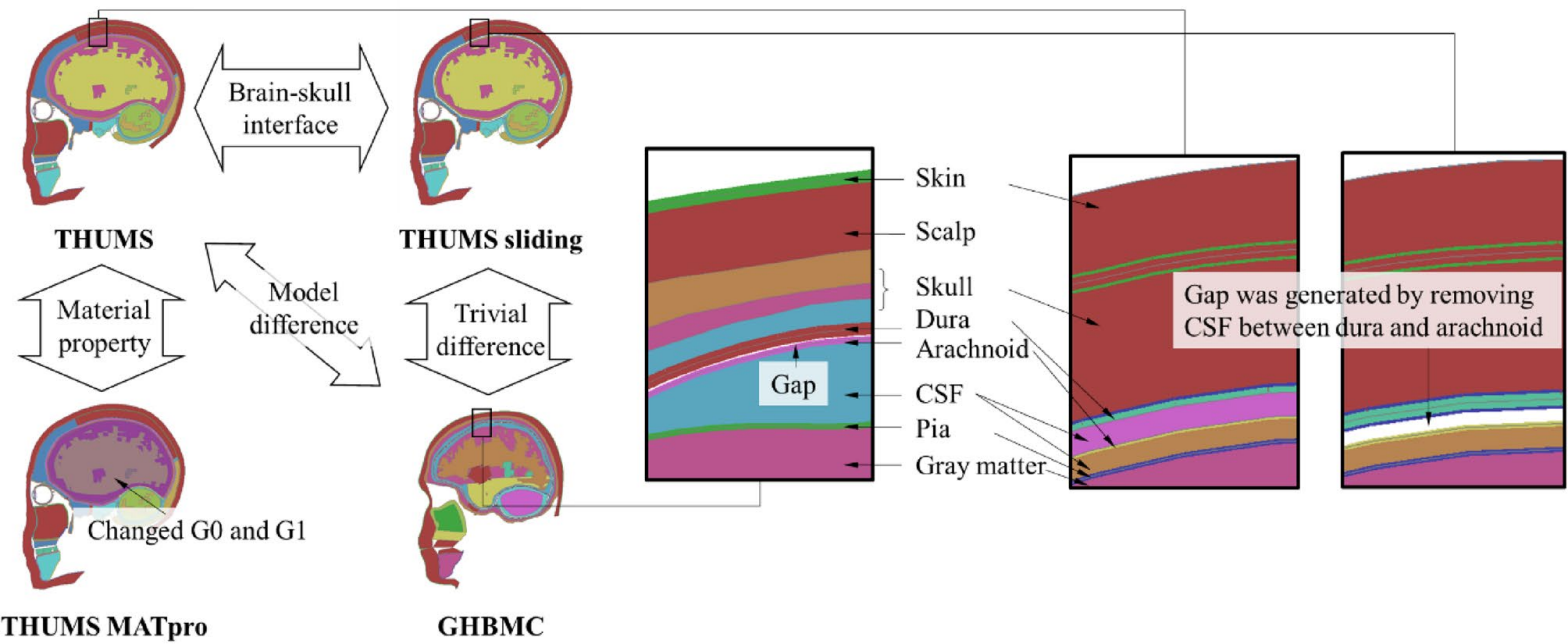
Sectional view (16.8 mm off sagittal plane) of the four head/brain models.

The material property of brain tissues and the interface modeling between the components are main aspects that might potentially influence on model performance in characterizing biomechanical and injury response under impact, and the mainstream models use different approaches and material property values. In this study, two additional approaches were assessed by changing the brain-skull contact and cerebrum material property of the baseline THUMS model. One was named “THUMS sliding” and generated by removing the CSF layer between dura and arachnoid in THUMS and changed it to sliding contact with the contact friction coefficient of 0.1. In fact, the sliding skull-brain interface in GHBMC was modeled this way. Another model, named “THUMS MATpro”, was generated by changing the material property in THUMS. The material property data of brain tissues are quite scattered in the literature. The material definition of gray matter and white matter in THUMS and GHBMC is linear viscoelastic. The bulk modulus (K), short-term shear modulus (G0) and long-term shear modulus (G1) are three main parameters for stiffness characterization. Comparing to the settings in other FE brain models, the values of G0 and G1 are relatively lower in THUMS and GHBMC. Hence, in the THUMS MATpro, G0 and G1 were adjusted to the values in SUFEHM (G0: 6000 Pa → 49,000 Pa; G1: 1200 Pa → 16,000 Pa), which are the highest values found in the literature. Figure 2 shows the four head/brain models for investigating the influence of brain-skull interface and material property on injury characterization. As THUMS, THUMS sliding, and GHBMC have same brain material property while THUMS and THUMS MATpro have same skull-brain boundary, the influence of different aspects of modeling approaches can be investigated by comparing different parts of models, as marked in Fig. 2.

### Load cases and animal tests for assessing DAI and ASDH

To evaluate model performance in addressing typical brain injuries, animal tests from literature were selected. One reason is that the numbers of cadaver tests and human in vivo tests are limited. Another reason is that some injury symptoms could only be observed in vivo test, for example, diagnosis of DAI needs to observe heart rate changes and unconsciousness. By reconstructing load cases and injury outcomes of animal tests, we can use the injury results from the tests to assess the injury prediction ability of injury metrics in different head/brain models.

**Figure 3:**
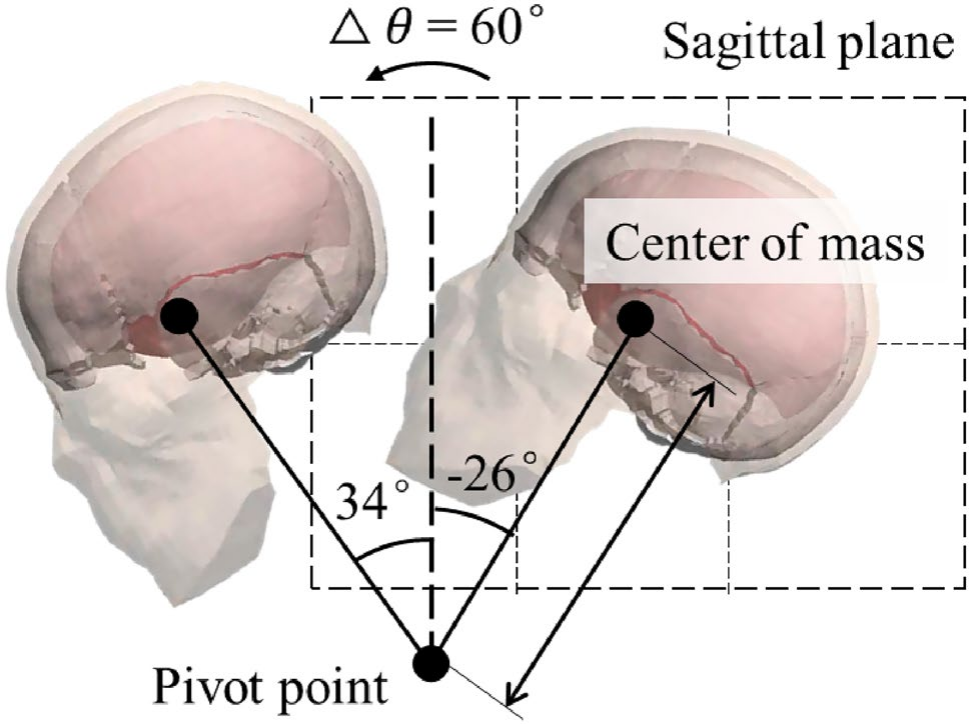
Loading mode in Abel’s animal tests.

Many animal tests were performed on monkeys. Abel et al.^42^ performed rotational test on 40 rhesus monkeys. Stalnaker et al.^43^ used 39 monkeys and 2 cadavers, and Nusholtz et al.^44^ used 3 live anesthetized and 3 postmortem rhesus monkeys and 9 cadavers. The 40 loading conditions in Abel’s tests were used in this study as classification between injury and non-injury (ASDH and DAI). In Abel’s tests, the head motion was constrained mechanically to achieve control and reproducibility of the physiological and pathological consequences. Sagittal plane rotation about a fixed axis was employed to the monkeys’ head due to simplicity of the kinematics of plane motion and existence of the previous investigations. As a result, the rigid body motion of any point in the head could be determined from the angular acceleration, $\ddot{\theta }$, and the distance from the point to the axis of rotation, *r*. For the series of experiments reported in the literature, the angular displacement was 60° in each case. The head of the supine animal started from rest in an extended position, $\theta$= − 26°, and was accelerated and then brought to rest at $\theta$= 34°. Peak values of angular acceleration ranged from 1.8 × 10^4^ to 1.2 × 10^5^ rad/s^2^ (Fig. 3).

**Figure 4:**
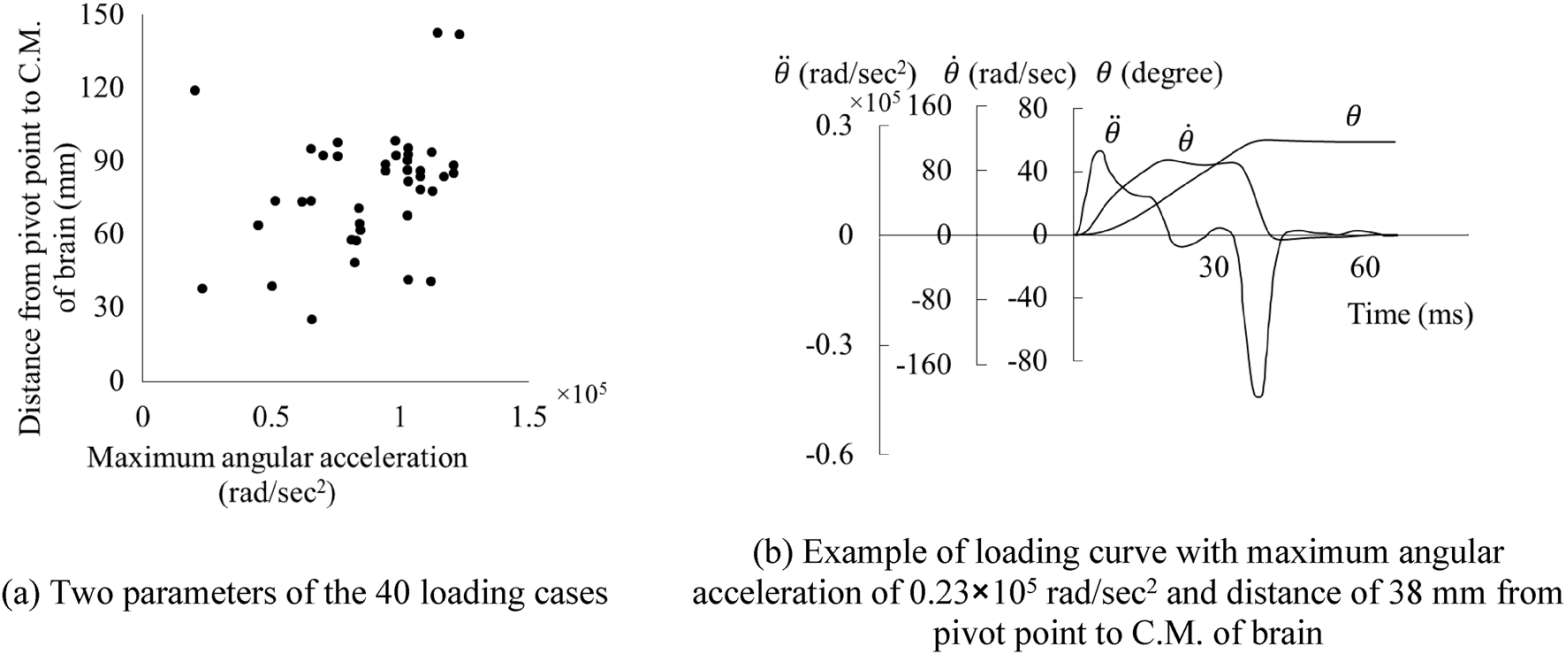
The 40 load cases and loading curves in Abel’s test^42^.

Abel’s tests had 40 loading conditions with various peak values of angular acceleration ($\ddot{\theta }$), and distance from pivot point to C.M. of brain (*r*). The waveshapes of the acceleration curves in the tests were similar (Fig. 4). To transfer the loading condition from animal tests to human FE simulations, the kinematic loading conditions were scaled in amplitude and time to satisfy the equal stress/velocity scaling relationship, i.e., translational velocity scaled by 1, angular velocity by 1/*λ*, and time scaled by *λ*, where *λ* is the scaling ratio. *λ* equaled 2.47 for the rhesus monkeys^10^. With the 40 load cases and the 4 FE brain models, in total 160 head impact cases were simulated using SMP Ls-dyna R6.1.2 and 28 CPUs per case.

### Selection of injury metrics

In this study, when evaluating brain injury risks, we used the stress/strain-based injury metrics, as such metrics are local-focused and quantitative. The biomechanical and deformative response of brain tissue at specific sites can be characterized with the metrics in a definitive manner. It is therefore intuitive and straightforward to make either inter-subject or intra-subject comparison.

The maximum principle strain and maximum principle stress are two important indicators. They can directly reflect the loading intensity and mechanical response of the models. Over the last half century, scientific evidence has indicated that most traumatic brain injury were caused by mechanical failure of brain structure, such as axonal damage^5,6^. Consequently, to a certain extent, the maximum principle strain and maximum principle stress can characterize the probability of brain injury, and such a use has also been continuously discussed and improved^10,41^. As the cerebrum in THUMS and GHBMC were all modeled with constant stress solid elements, the maximum principal stress and maximum principal strain of each cerebrum elements may be conveniently calculated with the stress and strain tensors from simulation output files (we wrote a MATLAB code to facilitate the data readout and processing though). CSDM, a strain-based injury metric introduced by Bandak and Eppinger^45^, monitors the accumulation of strain damage by calculating the volume fractions of the brain experiencing strain levels greater than various specified levels. CSDM was intended to reflect medical speculations of DAI. The correlation between DAI and CSDM was analyzed by Takhounts et al.^10^. Thus, CSDM, as an example of the brain injury indicators, was calculated and discussed as well.

### Ethical approval and consent to participate

No live human/animals experiment was performed in this study, as the human/animals experimental data was from literature.

## Results

We will show and analyze the results in three aspects. The first aspect is comparison of kinematic response and strain and stress distributions in brain between the four models, finding out the effects of the modeling approaches. Then the injury metrics of the model outputs were compared. Lastly, the brain injury results from Abel's test were compared with the simulation results to assess the injury characterization capability of the four models.

### Kinematic response and strain and stress distribution

**Figure 5:**
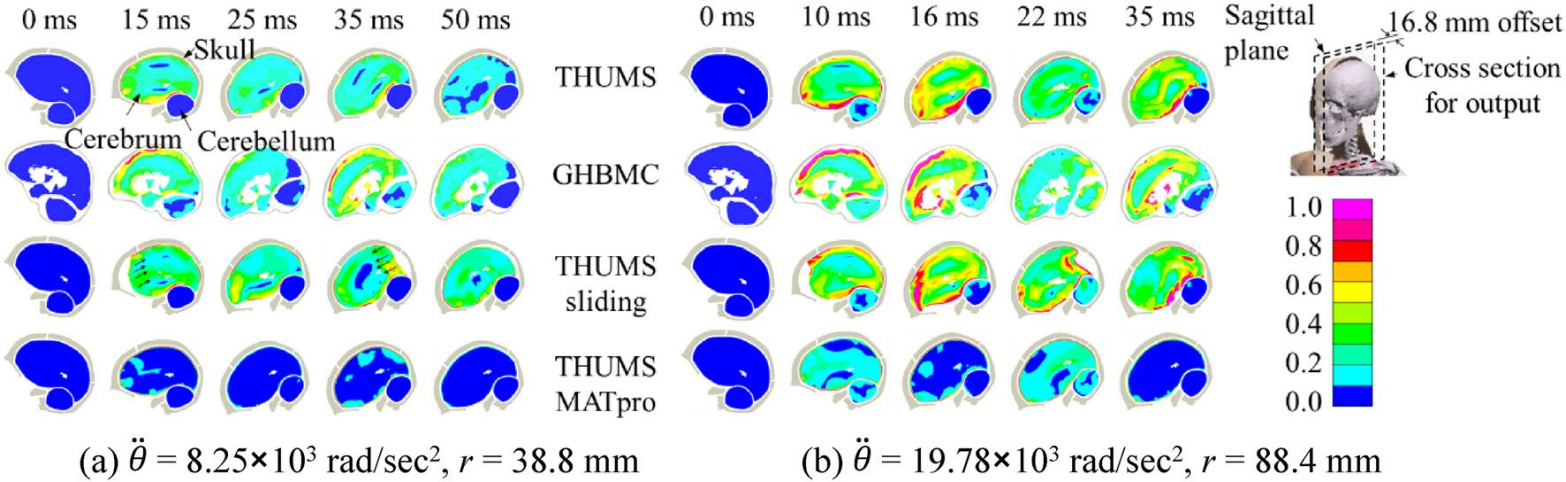
Kinematic response and first principal strain contour plot in two representative simulation cases.

The peak angular acceleration in Abel’s^42^ animal tests varied from 3.34 × 10^3^ to 20.16 × 10^3^ rad/s^2^ after scaling. Taking two representative load cases as example, a mild one and a severe one, Fig. 5 shows the kinematic response and the first principal strain in cerebrum and cerebellum in simulation. To avoid the location of falx cerebri, the output was calculated on a cross section 16.8 mm offset from the sagittal I plane. The case of relatively mild loading had peak angular acceleration of 8.25 × 10^3^ rad/s^2^ and rotation radius of 38.8 mm (Fig. 5a), and under the loading, the monkey in Abel’s test did not suffer ASDH or DAI. The THUMS sliding model gave the largest movement because of the sliding gap between dura matter and arachnoid matter. During the first 20 ms, the brain was accelerated in the counterclockwise direction, causing a relative motion to the posterior skull. After 20 ms, the brain was suddenly decelerated, causing the cerebrum tissue hitting the anterior skull due to the inertia. The maximum principal strain appeared at the surface of cerebrum, especially at the contact surface with the cerebellum in THUMS. The strain level in the THUMS MATpro model was the lowest among the four models, as the shear modulus of cerebrum was set 5–10 times larger than that in the THUMS original model. The case of relatively severe loading resulted in peak angular acceleration of 19.78 × 10^3^ rad/s^2^ and rotation radius of 88.4 mm (Fig. 5b). Under the loading, the monkey suffered both ASDH and DAI, and the maximum principal strain was significantly larger than that in the case of relatively mild loading.

**Figure 6:**
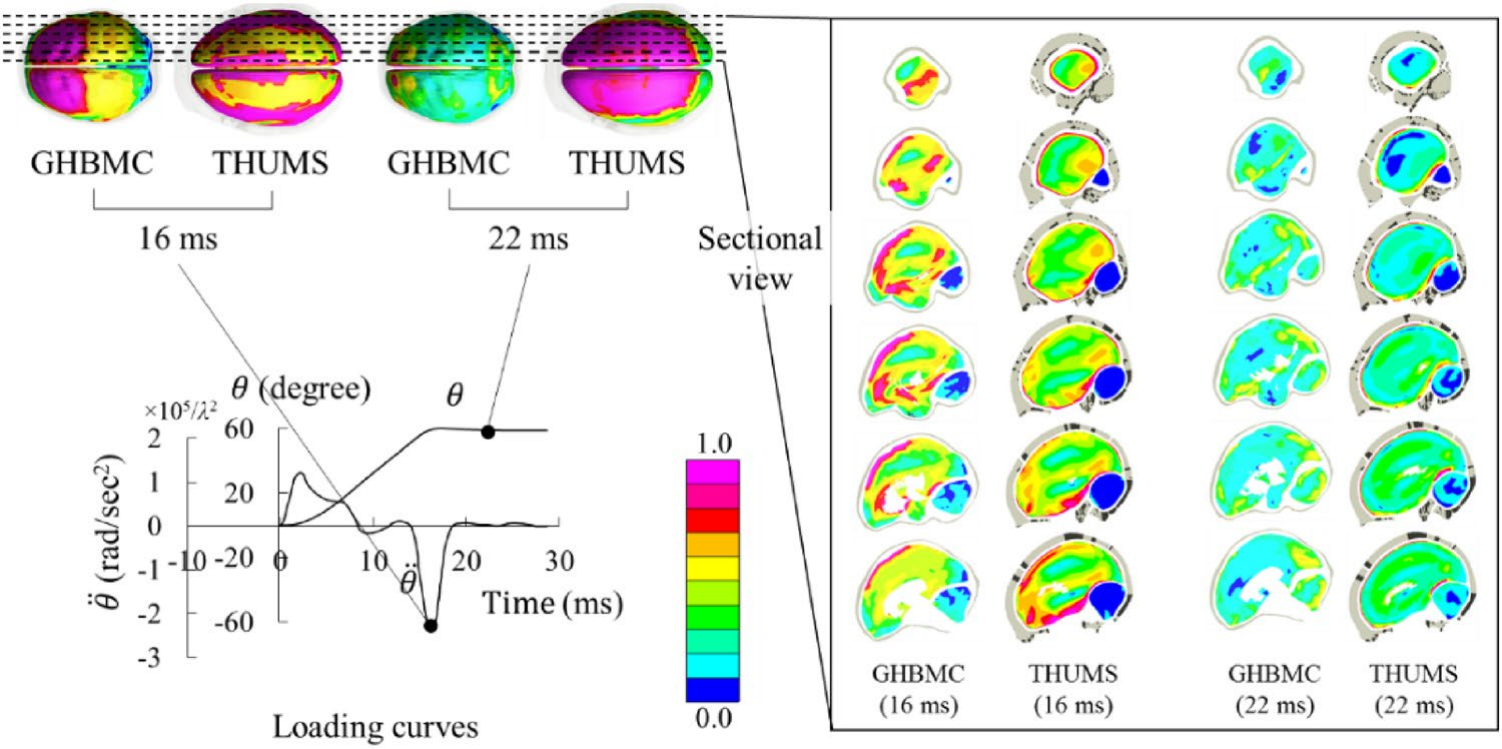
First principal strain distribution in THUMS and GHBMC under the relatively severe loading condition.

The strain distributions in brain in THUMS and GHBMC were different, mainly caused by different skull-brain contact definitions. Figure 6 shows several sectional strain plots under the relatively severe loading at two representative timings (16 ms and 22 ms). 16 ms was the time when the deceleration curve peaked and the maximum strain occurred. All the surface areas in the THUMS brain at that time had high strain levels. In contrast, the brain of GHBMC sustained a high local strain state in the anterior area. The six sectional strain plots with equal interval show the internal strain condition of the brain. In contrary to the distributed high strain state on the brain surface, the high strain area inside the THUMS brain is highly localized. Only the solid elements on the first layer of the surface sustained high strain, with thickness less than 3 mm. In other words, significant amount of energy was absorbed by elements on the surface that connected the brain and the skull, and the deformation did not spread much into the internal area. The strain distribution in GHBMC was different from that in THUMS. Its high strain state on the surface spread to the internal cerebrum and appeared at the frontal top part of cerebrum and near ventricle.

**Figure 7:**
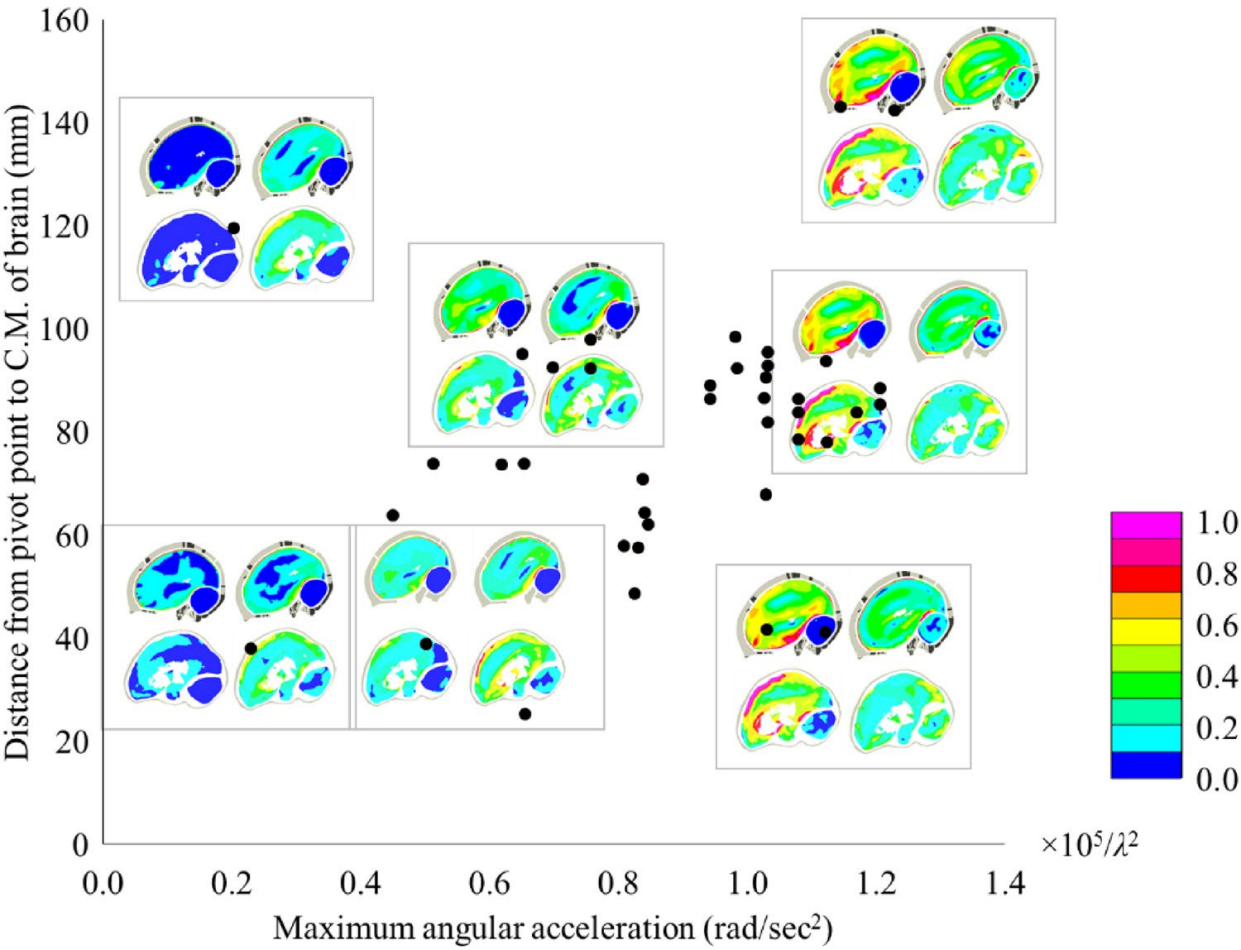
The first principal strain contour plots of THUMS and GHBMC under seven loading conditions (small figure: top—THUMS, bottom—GHBMC, left—maximum angular deceleration, right—brain rebounding in skull).

In addition to the modeling approaches, the loading conditions affect the stress and strain output too. In Abel's tests, the loading mode was relatively simple, which was a one-directional rotation including an acceleration phase and a deceleration phase. The loading condition can be characterized with two parameters, the rotation center position and the maximum angular acceleration. Figure 7 shows the first principal strain contour plot of THUMS and GHBMC in seven loading conditions listed in a coordinate system, the horizontal and vertical coordinate value of which represented the corresponding loading severity and loading form in Abel’s tests. The brain models in THUMS and GHBMC both had a larger strain distribution on cerebrum under loading with larger maximum angular acceleration. Compared with the maximum angular acceleration, the distance from pivot point to C.M. of brain had very limited effect to the strain magnitude, especially when the maximum angular acceleration was large.

### Injury metrics output

**Figure 8:**
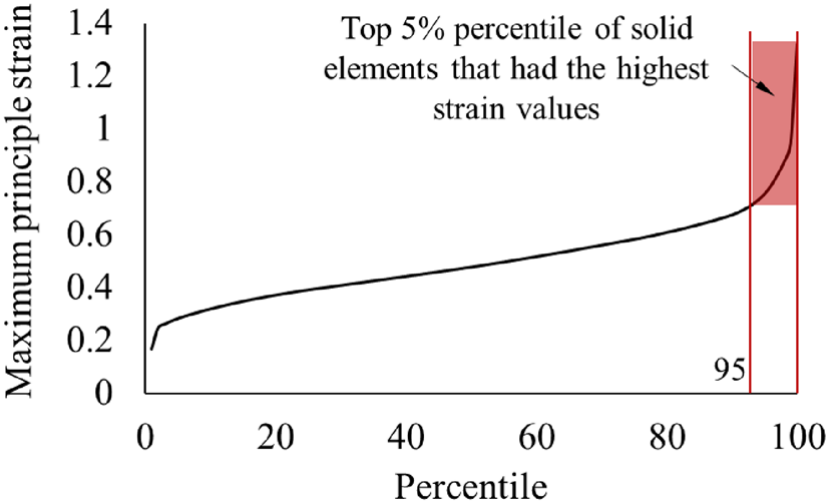
The Maximum Principal Strain of all the solid elements of the cerebrum in THUMS in the entire time domain under the second loading condition.

The cerebrum parts in THUMS and GHBMC brain models have 23,020 and 47,323 solid elements, respectively. Their strain and stress tensors were outputted and the first principal strain and the first principal stress were then calculated. Throughout the entire time domain, the maximum first principal strain in of all the solid elements were identified, and then the maximum strain values were ranked in percentile groups of the numbers of the solid elements. Upon such a scheme, for the second loading condition shown in Fig. 5, Fig. 8 shows the maximum first principal strain levels versus the percentile of the solid elements of cerebrum in THUMS. The strain level in the top 5% percentile group of the solid elements that had the highest strain values increased significantly. The high strain state in this small portion of the elements was caused by the local force concentration and the discreteness of the numerical simulation. The maximum strain values exhibited large randomness and no regularity when comparing between the models under different loading conditions, which means, they cannot represent the strain output performance of the models. Consequently, to get rid of the problem from the few elements that had very high amplitudes and for better comprehension of difference among the models, in this study, the maximum strain and stress were calculated by ignoring the five percent elements that have the highest values. (Note: The maximum principal strain is usually abbreviated to MPS in literatures. In order to prevent it from being confused with the maximum principal stress, the full name is used in the following.)

**Figure 9:**
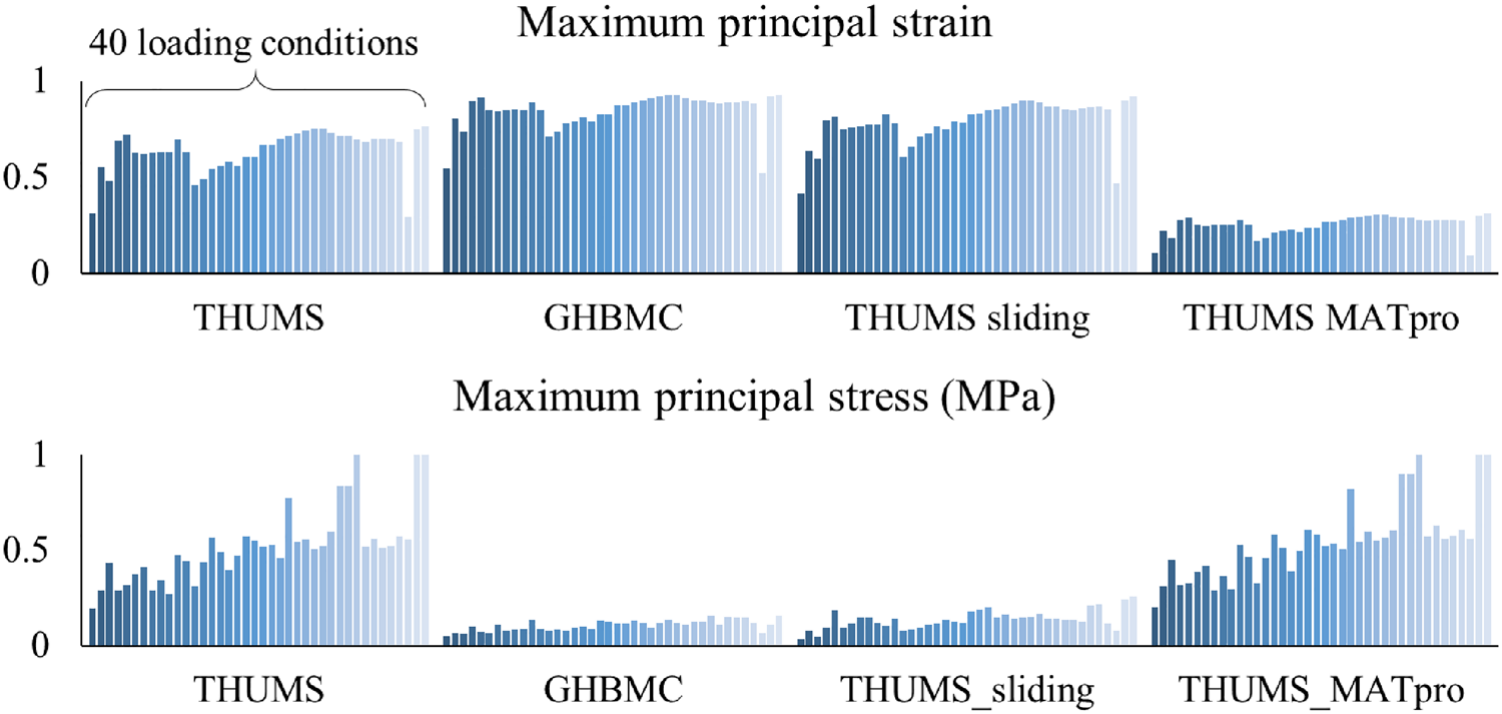
The maximum principal strain (top) and maximum principal stress (bottom) of the cerebrum in the 160 cases.

The maximum principal strain and maximum principal stress of cerebrum in the 160 cases are shown in Fig. 9. Among the cases, strain and stress distributions between the four models have similar shapes, indicating that the loading severity can be similarly reflected through stress and strain values in the four models. The mechanical output of different models under the same loading condition is different. The maximum principal strain of cerebrum in GHBMC is larger than that in THUMS, as the brain surface in THUMS is connected to the skull and it limits the deformation of the cerebrum. After changing the skull-brain connection in THUMS to sliding contact, such generated THUMS sliding model shows a larger maximum principal strain, similar to the GHBMC output. The THUMS MATpro model has the lowest maximum principal strain among the four models as its shear modulus is set 5 ~ 10 times larger than that in the other models. The difference between the maximum principal stress of the THUMS and GHBMC is large, compared to the difference in strain. The sliding contact definition between the skull and brain in GHBMC releases the high stress state at the interface, and the maximum principal stress is about one fifth of that in THUMS. The THUMS sliding model have similar stress magnitude compared with GHBMC. The maximum principal stress in the THUMS MATpro model is the same as that of THUMS, indicating that the shear modulus in brain does not affect the stress output much.

**Figure 10:**
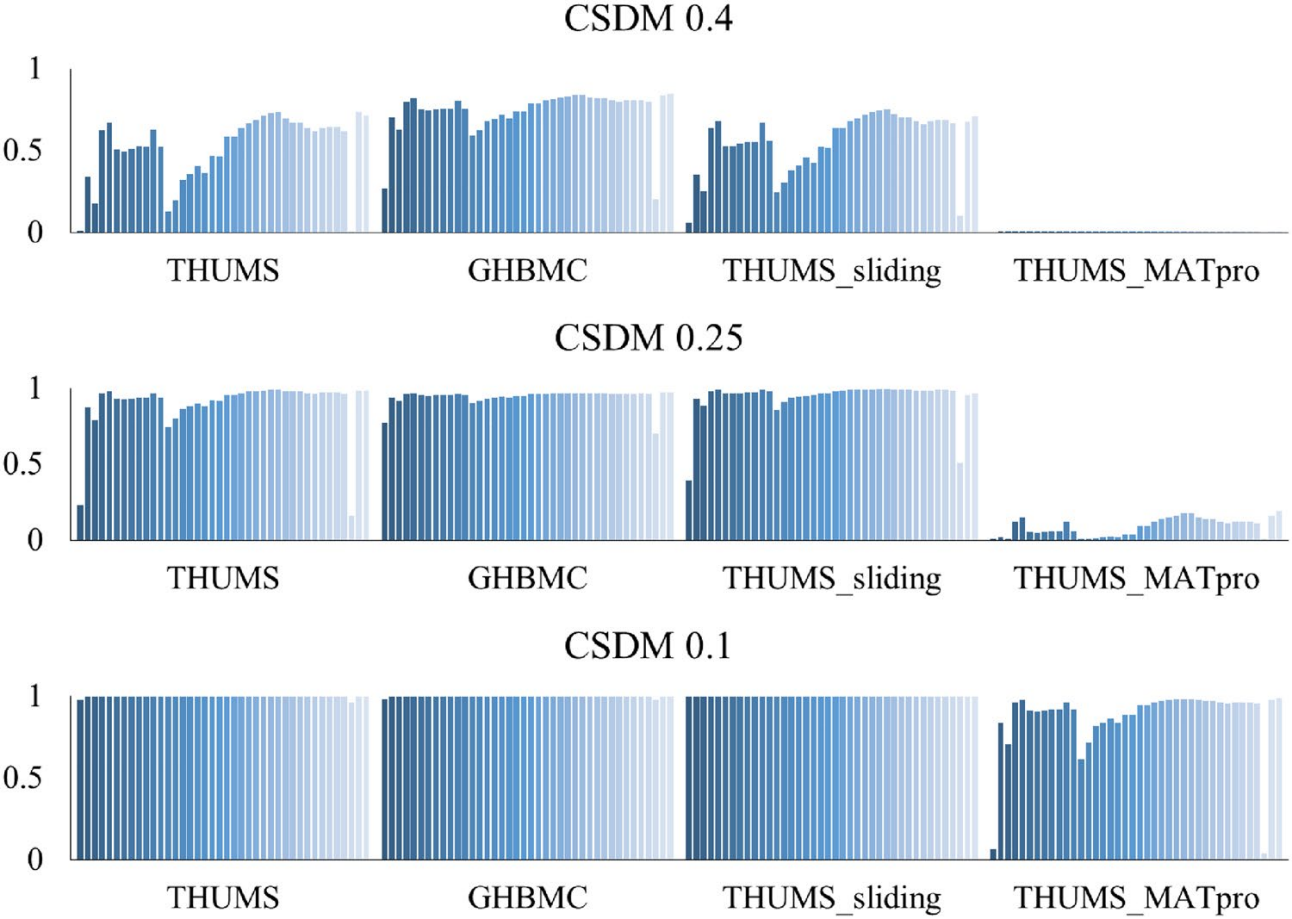
CSDM of the four brain models among the 40 loading conditions.

In the following, CSDM with specified strain levels of 0.1, 0.25, 0.4 were discussed, as shown in Fig. 10, covering the levels in most previous research and the injury threshold of axon observed from previous experiments. The CSDM 0.1 (0.1 here means the specified strain level for CSDM calculation was set to 0.1) in THUMS, GHBMC, and THUMS sliding model in the 40 loading conditions were all very high and close to 1. The difference of CSDM among the cases was less than 0.04, and 109 of the 120 cases had CSDM equal to 1. At the other extreme, the CSDM 0.4 in the THUMS MATpro model among the 40 cases were all lower than 0.01, which also led to insignificant output differences among various loading conditions. Except for the listed cases, the difference of CSDM values was obvious under the different loading conditions. In general, as the CSDM was calculated based on the maximum principal strain, the differences between the four models were similar when comparing the strain. The THUMS MATpro model had the lowest value, while GHBMC had the highest. CSDM in the THUMS sliding model was higher than that in THUMS as the skull-brain interface was set to a greater freedom.

### Comparison of injury characterizing capability

**Figure 11:**
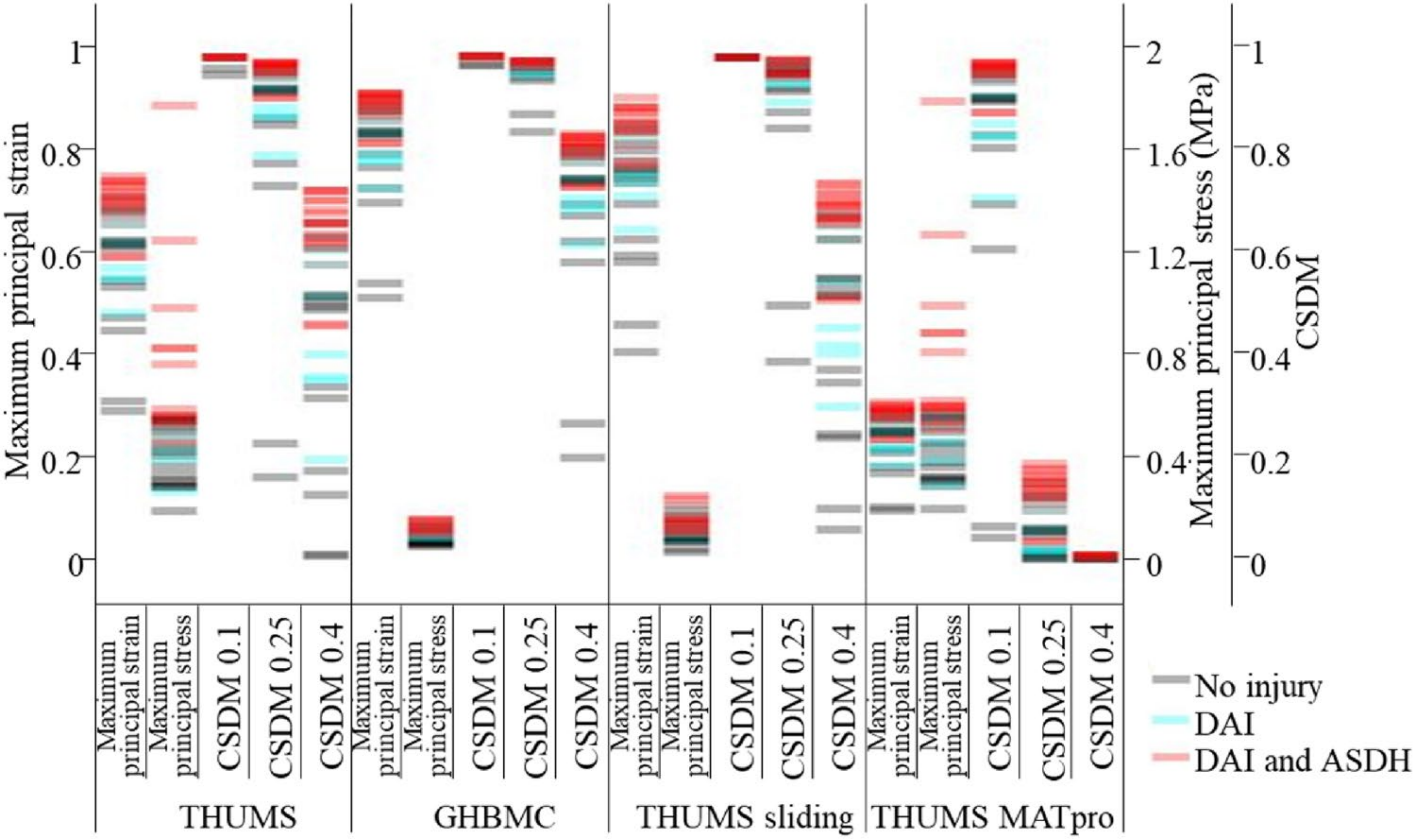
Injury metric values of the four brain models under the 40 loading conditions.

Abel’s experiments provided the injury results of every loading conditions on brain. Two injury types, ASDH and DAI, were observed in the experiments. Figure 11 shows the injury metric values of the four brain models under the 40 loading conditions. The colored circles marked the injury results of the 40 loading conditions from Abel’s tests using monkeys. It needs to be mentioned that no monkeys suffered ASDH without suffering DAI in the tests, so the injury results were divided into three categories, “No injury”, “DAI”, and “DAI and ASDH”. It can be seen from the color distribution that the maximum principal strain and the maximum principal stress resulted in a clear distinction between the injured and uninjured cases. The CSDM values varied greatly among the four models. THUMS, GHBMC, THUMS sliding seems to be more suitable for CSDM with higher levels (0.25 and 0.4) and THUMS MATpro were more suitable for CSDM with lower levels (0.1 and 0.25).

In order to describe and understand the applicability of different injury metrics in the four models, logistic regressions were performed based on the experimental results for those metrics^10,46,47^. We used p-value and *χ*^*2*^ to represent the fitting degrees of the logistic regression, as shown in Table 2. Most regressions were acceptable with p-value lower than 0.001, while a small number of regression cannot be completed since the correlation between the calculated injury metric and the injury results was poor. For example, when regressing the injury results (DAI and ASDH) using CSDM 0.1 in THUMS, the data points were so clustered that the maximum likelihood estimates of the parameters do not appear to exist.

**Figure 12:**
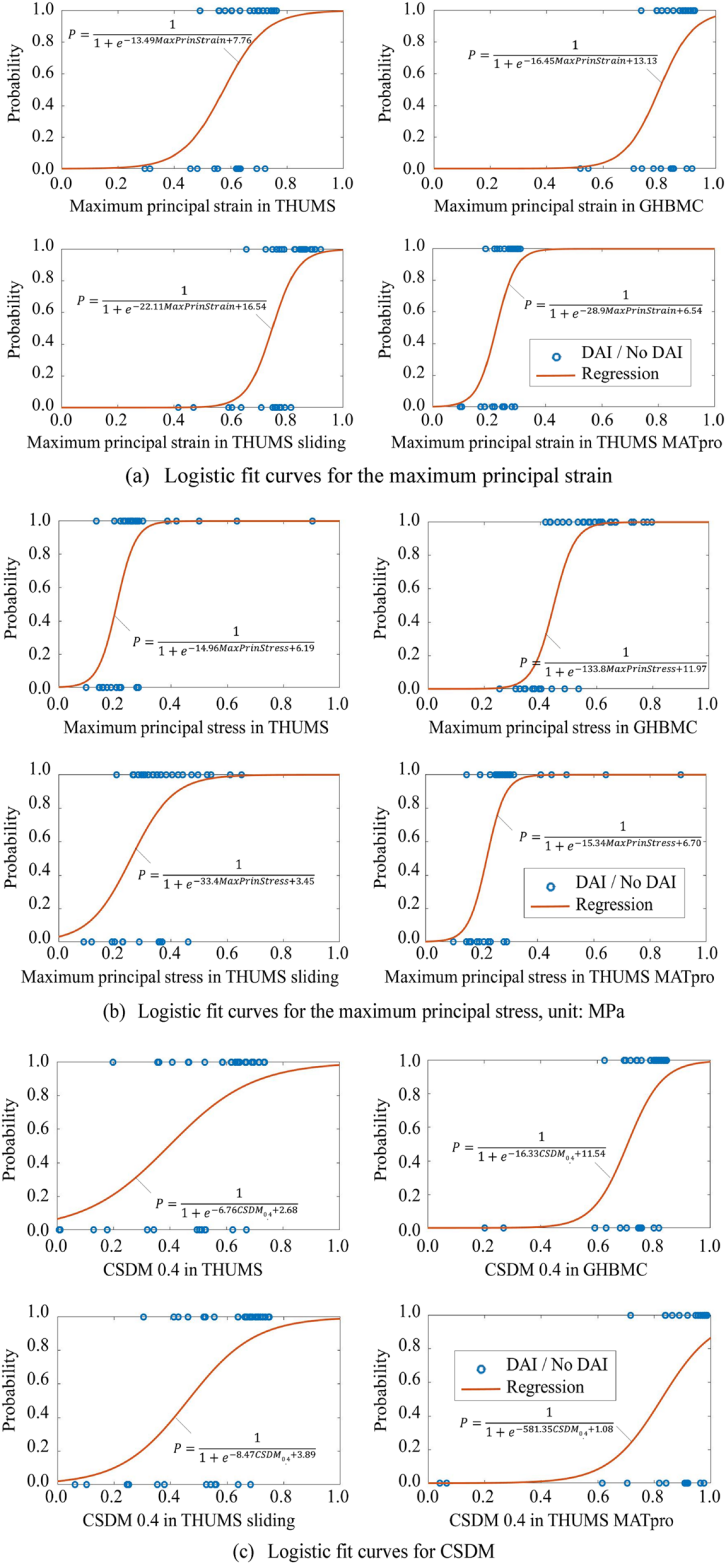
Logistic fit curves in the four brain models.

Each logistic regression could be represented with an s-shaped curve, showing the probability of DAI or ASDH with injury metric values. Some representative regression curves for DAI are shown in Fig. 12. The regression functions are marked in the figure, and they can be used to predict injury risk for researchers using various brain models. A quick observation was that the s-shape curves calculated using different models for the same indicator were different. More specifically, for example, the established maximum principal strain value corresponding to 50% probability of DAI in THUMS was much smaller than that in GHBMC and THUMS sliding but higher than that in THUMS MATpro. Referring to the p-value discussed in Table 2, those regressions were all reasonable and acceptable but leading to different regression results. This difference was most obvious in regression with CSDM. When using 0.1 as a specified strain level for CSDM calculation, the CSDM values in THUMS, GHBMC, and THUMS sliding under the 40 loading conditions were all close to 1. This data cluster made it hard to effectively distinguish between injured and uninjured conditions and unable to regress the s-shape curves. However, the CSDM in THUMS MATpro regressed the injury results well, especially for predicting the ASDH (p-value < 0.001, *χ*^*2*^ = 21.27).

**Table 2:** P-value and *χ*^2^ in logistic regression.

	THUMS					GHBMC				
	Maximum principal strain	Maximum principal stress	CSDM 0.1	CSDM 0.25	CSDM 0.4	Maximum principal strain	Maximum principal stress	CSDM 0.1	CSDM 0.25	CSDM 0.4
**DAI**										
p-value	0.0005	0.0000	–	0.0008	0.0006	0.0011	0.0000	0.0038	0.0004	0.0005
*χ*^2^	12.13	19.01	–	11.22	11.68	10.73	30.61	8.36	12.34	12.29
**ASDH**										
p-value	0.0000	0.0000	–	0.0000	0.0000	0.0000	0.0000	–	0.0000	0.0000
*χ*^2^	23.27	27.58	–	22.28	23.52	21.63	28.96	–	24.22	24.13

	THUMS sliding					THUMS MATpro				
	Maximum principal strain	Maximum principal stress	CSDM 0.1	CSDM 0.25	CSDM 0.4	Maximum principal strain	Maximum principal stress	CSDM 0.1	CSDM 0.25	CSDM 0.4
**DAI**										
p-value	0.0000	0.0006	–	0.0006	0.0002	0.0007	0.0000	0.0011	0.0009	0.0007
*χ*^2^	20.12	11.86	–	11.63	13.70	11.55	20.90	10.59	11.06	11.37
**ASDH**										
p-value	0.0000	0.0026	–	0.0000	0.0000	0.0000	–	0.0000	0.0000	0.0000
*χ*^2^	31.16	9.08	–	17.44	24.84	22.47	–	21.27	22.17	23.18

## Discussion

This section is devoted to more detailed comparisons and mechanism analysis.

### Influence of modeling approaches on kinematic and mechanical response of brain

Large differences were found between THUMS and GHBMC in the maximum principal stress and strain under the loading conditions in Abel’s monkey tests. The differences were caused by the different contact definitions of the skull-brain interface. First of all, the two models were very similar in model composition and material property. Only the contact definition between dura and arachnoid was significantly different (Table 1). The relatively strong connection between the brain surface and the skull in the THUMS model and the sliding contact in the GHBMC model represent the two typical interface definitions. In the skull-brain system, the deformation of the skull is negligible compared to that of the brain, and the skull can be regarded as the boundary that causes the movement and deformation of brain.

**Figure 13:**
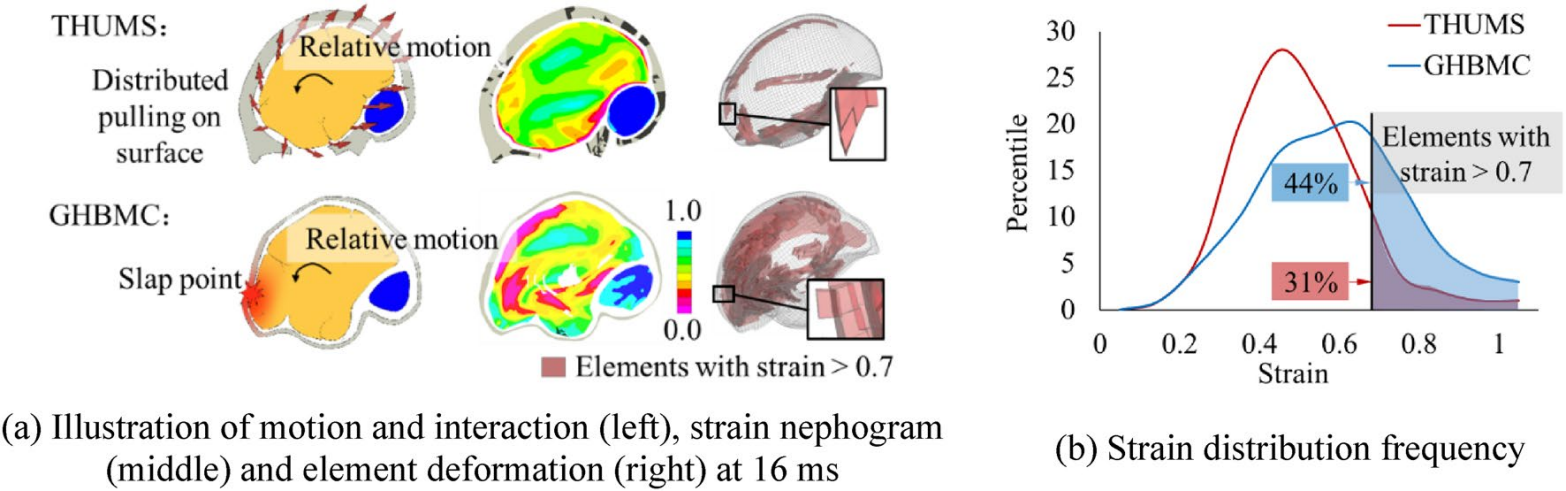
Comparison of motion and interaction between two skull-brain interface definitions under the severe loading condition.

In THUMS, the skull-brain is connected and constrained through the CSF layer. Although the CSF’s material modulus is much lower than that of the brain, the connection and constraint are stronger than the constraint with sliding, which is only constrained along the tangential surface with a low friction coefficient. When the skull is accelerated and/or decelerated rapidly on the sagittal plane, the surface of the brain follows the movement of the skull and then pulls its interior part to accelerate or decelerate (Fig. 13a). The delay of brain’s motion due to its inertia results in large shear deformation at the brain boundary. If the brain elements in the model are assigned low stiffness material properties measured in the experiment, the elements in the first layer next to the boundary are greatly deformed. On one hand, this large deformation "protects" the internal elements to a certain extent and reduces the overall deformation of the interior part. On the other hand, the strong connection between skull and brain also increases the stress level. Most elements with higher strain levels are located on the surface of brain and exhibit obvious shear deformation (Fig. 13a).

In the GHBMC brain model, the sliding contact defined in the skull-brain interface made it difficult to directly transmit the acceleration from the skull to the brain through the interface. Consequently, the resulted acceleration of the brain is due to slapping (pushing) the brain surface by the inner wall of the skull (Fig. 13a). In this case, the high-strain areas of the brain are naturally gathered near the "slapping point" and extended from the surface to the internal brain (Fig. 6). Compared to the THUMS brain model, the lack of strong constraints at the interface makes the brain of GHBMC relatively free to deform, resulting in higher strain and lower stress (Fig. 6). The elements with higher strain in GHBMC are more than those in THUMS, spreading out from the surface to the interior of the brain, exhibiting obvious compressive deformation (Fig. 13a). Figure 13b shows the maximum principal strain distribution frequency of all solid elements in brain of THUMS and GHBMC. The solid elements with the maximum principal strain greater than 0.7 account for 44% in GHBMC, while only 31% in THUMS. This finding is consistent with the previous mechanism analysis and observation from the sectional plots.

In the THUMS sliding model, only the skull-brain connection form was changed from the direct attaching to sliding contact, making it similar to the boundary definition in GHBMC except some trivial differences. As expected, the THUMS sliding model gave similar stress and strain output to that of the GHBMC model. It revealed that the difference between THUMS and GHBMC in strain and stress output was mainly caused by the difference in the skull-brain connection.

As shown in Fig. 5, the brain displacement of THUMS sliding was found much larger than that of THUMS and GHBMC, which was similar to the result by Wang et al.^32^. In that study, four kinds of skull-brain connection were defined, from frictionless sliding contact to fully constrained connection, and the relative displacement of the brain with sliding interface was found much larger than that of the original THUMS model. In this study, we went further to analyze the resulted strain distribution in brain and assessment of model's ability of injury characterization. In THUMS, when replacing the CSF constraint between dura and arachnoid with sliding, a gap of 0.8 mm was formed due to the original thickness of the removed CSF layer. In comparison, the gap between the skull and the brain in GHBMC was 0.1–0.2 mm (Fig. 2). The greater gap in the THUMS sliding model increased the freedom of brain movement, so the displacement of the brain in THUMS sliding was even larger than that of GHBMC that has a similar skull-brain interface definition. Furthermore, in the GHBMC model, 11 pairs of bridging veins were defined using beam elements connecting the SSS and the cerebral pia mater. These veins were modeled as beams with outer diameter of 2.76 mm, inner diameter of 2.73 mm, and Young’s modulus of 30 MPa^48^. It strengthens the connection between the skull and the brain and reduces the relative movement between them.

**Figure 14:**
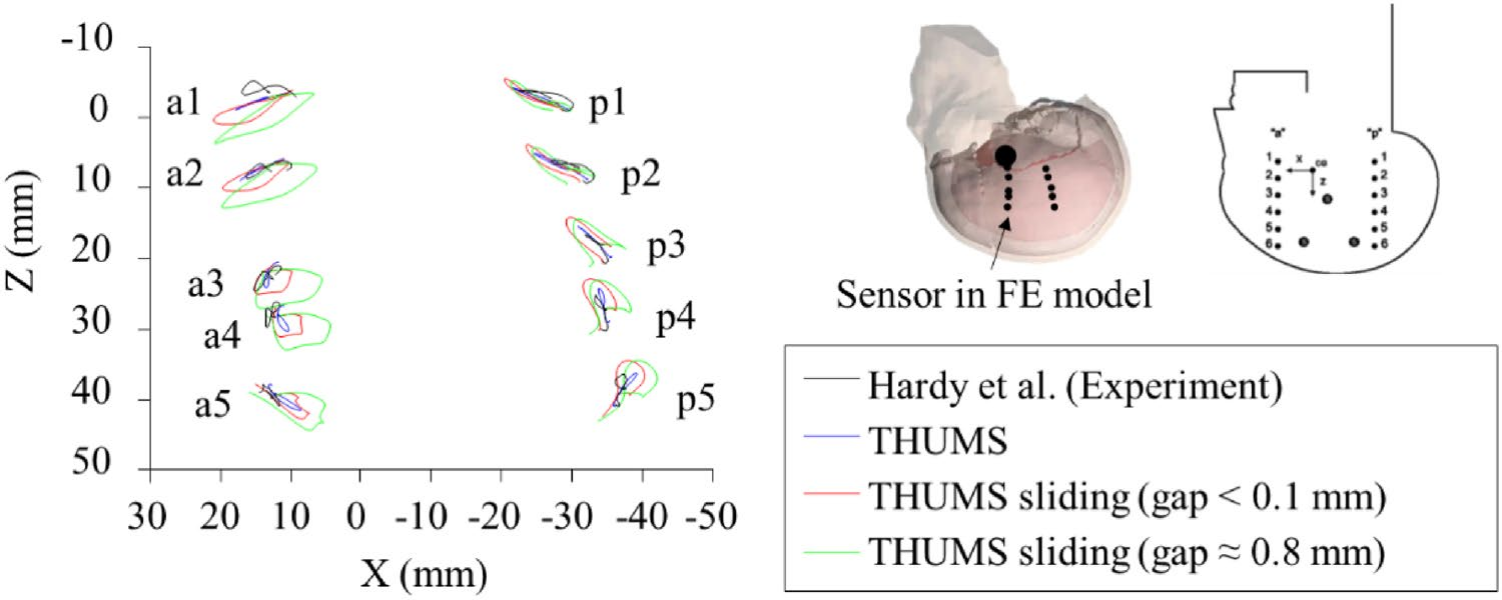
Comparison of the NDT trajectories predicted using THUMS model when changing the brain-skull interface modeling approach and experimental results by Hardy et al.^36^.

To assess whether the gap distance could affect the brain’s mechanical response, we constructed different gap distances on the sliding interface in the THUMS brain model, and compared the brain motion by reconstructing Hardy et al.^36^’s cadaver test. In Hardy’s test, the trajectory of the neutral density targets (NDT) implanted in the brain was used to observe the movement of the brain relative to the skull. The loading conditions on the head were similar to the Abel’s experiment, with a larger movement along the sagittal axis. In finite element simulation, sensors were defined at the same location with the NDTs. Figure 14 shows the relative displacement of the NDTs in brain relative to the skull in THUMS, two THUMS sliding with different gap distances, and Hardy's test. The relative motion in THUMS was the closest to Hardy’s test, which can also represent it in GHBMC under the same condition, as the two models have both been validated with the same loading condition before. The relative motion of the THUMS sliding model was larger than that of THUMS and Hardy’s test. The larger the gap is, the greater the relative motion is. On the other hand, the stress and strain output of the models with the two gap distances were very similar. For example, the maximum principal stress in the brain of the two THUMS sliding models (0.15 MPa for gap < 0.1 mm; 0.10 MPa for gap = 0.8 mm) were far less than that of THUMS (0.53 MPa). In result, there are significant differences in mechanical responses between sliding and no-sliding, while not much difference between sliding gap distance varying from 0.1 to 0.8 mm.

### Applicability of injury metrics with different brain models

In the past few decades, researchers have developed different brain models to characterize and predict brain injuries. Correspondingly, some brain injury metrics were established based on cadaver tests or animal tests, and correlated with different brain injuries by regression. There were also research on the correlation between different injury metrics (Fig. 1). This paper emphasizes that critical characteristics of the models need to be considered when using injury metrics. In other words, differences in modeling approaches may affect mechanical responses as well as strain and stress distributions in brain. Since the injury metrics are mostly strain or stress based, the modeling approaches would consequently affect applicability of injury metrics. The two papers by Takhounts^10,41^ already discussed this issue. Using different versions of the SIMon model, Takhounts studied correlation between RMDM and ASDH and obtained contradictory conclusions that were attributed to the model difference. In fact, similar issue was seen when studying modeling approaches and injury metrics on other body parts as well. The brain modeling is just more complex.

In this study, Abel's animal test was reconstructed using the four models under study, and the injury results were used for regression of the injury metrics. We found that the injury metrics calculated from the four brain models had different degrees of correlation with the injury results. This is somewhat similar to the findings by Takhounts^41^. In addition, the difference in degrees of correlation is not directly related to the output value of injury metric. For example, although the THUMS sliding model had stress output similar to that of the GHBMC model, which were much smaller than that of the THUMS model, the regression correlation between the maximum principal stress and ASDH of the THUMS sliding model (p-value = 0.0026, *χ*^2^ = 9.08) was far worse than that in the THUMS model and the GHBMC model (p-value < 0.0001, *χ*^2^ = 27.58 and 28.96). However, it did not mean that the THUMS sliding model was much worse than the THUMS or the GHBMC models. If using the maximum principal strain as injury metric to regress DAI, the performance of the THUMS sliding model was better than the other two models.

Furthermore, when using CSDM as injury metric, model with greater brain stiffness was more suitable for a lower strain level for CSDM calculation, and model with lower brain stiffness was more suitable for a higher strain level one. Moreover, the s-shaped logistic fitting curves of same injury metric were different between the four brain models. When using maximum principal strain to predict ASDH, the regression correlations of the four models were all good (p-value < 0.0001), but the 50% probability of ASDH corresponded to different maximum principal strain values in different brain models (0.67 for THUMS; 0.87 for GHBMC; 0.82 for THUMS sliding; 0.27 for THUMS MATpro). These results means that, the same value of injury metric in different brain models may mean different injury severity, and the same injury probability in different models may correspond to different values of injury metric.

### Limitations

In this study, the load cases for correlation, the selection of injury metrics and the diversity of size of the head/brain models are limited.

First, the loading condition selected from the tests by Abel et al.^42^ was somewhat single-mode, only representing inertial loading of head rotation along the sagittal axis. In real world accidents such as vehicle collisions, falls or assaults, the loading conditions on head and brain are more diverse.^25,26^) found that the impact direction will influence the relationship between kinematics and brain strain. Medical research has shown that falx cerebri would control the relative motion of brain under lateral impact loading and reduce the injury severity of brain. Hernandez et al.^49^ found that corpus callosum may be sensitive to coronal and horizontal rotations. The strain and stress distributions under lateral loading condition may be different from the analysis in this study.

Second, this study selected only three injury metrics for evaluation, which were the maximum principal strain, the maximum principal stress, and CSDM with various strain levels. Figure 1 shows many but not all injury metrics developed in the last half century. The validity of those injury metrics needs to be studied too. One step further, with the development of modeling of more detailed structures in brain, the influence of modeling approach to the output from those structures can be studied in the future, such as axonal strain^50,51^.

Third, this study used only one head geometry. The THUMS and GHMBC models were developed based on CT scan of the 50th percentile western male. Consequently, the four head models in this study only represent that of the averaged male. Influences of more head sizes, gender, age, ethnic, and other factors (e.g. brain disease) are not included in this study. In addition, the skull-brain interface definition and the brain material property evaluated in this study were relatively simple. More complex boundary definition forms and other material constitutive models are not reflected. For example, the material property definition of the CSF layer could affect the relative motion between the skull and the brain and its influence is worth of study.

## Conclusions

This study was aimed at discussing the influence of brain modeling approaches on model’s mechanical output and injury prediction. Four head/brain models based on THUMS and GHBMC with different modeling approaches were assessed. The following conclusions have been drawn.

1. The definitions of the skull-brain connection in head/brain models directly influence mechanical loading transmitted to the brain components as the brain follows skull’s motion. In the models where brain-skull interface connection is relatively strong, the acceleration of the skull is transmitted to the brain through the structural connection at the interface. It leads to stress and strain concentration on the surface of the brain. In contrast, if a sliding brain-skull interface is defined, brain’s acceleration is the result of slapping onto the skull or other surrounding components, and consequently, high-strain areas in the brain naturally gather near the "slapping point" and extend from the surface to internal areas of the brain. Although neither modeling approaches can fully reflect kinetic or kinematic characteristics of the skull-brain connection under impact or inertial loading, this study has shown that, to some extent, these models are useful in investigations of brain injuries when load cases are close to that in validation tests. Meanwhile, we need to recognize that brain materials and skull-brain connection are difficult to characterize, and therefore continuing research in this regard are necessary, for example, modeling constraint of the skull-brain connection to achieve more realistic transition from relatively strong skull to extremely soft brain tissue.
2. Compared to the fully connected interface, sliding interface slightly increases the maximum principal strain and significantly decreases the maximum principal stress. In addition, the stress and strain output are not sensitive to the different gaps set for the sliding interface.
3. The material property of the cerebrum influences the strain and stress under impact loading. Increasing its shear modulus will slightly increase the maximum principal stress and significantly decrease the maximum principal strain.
4. Injury metrics calculated from different brain models exhibit different degrees of correlation with the injury results from the tests. Model with greater brain stiffness is better correlated with lower strain level for CSDM calculation. When using different brain models, injury probability may correspond to different injury metric values.

## Data Availability

The datasets generated and analysed during the current study are available in the Open Science Framework repository, https://osf.io/x7wn2/?view_only=a6745deeb16047768dd99ecde89a392d.
